# Comparative Genome Analysis Reveals Metabolic Versatility and Environmental Adaptations of *Sulfobacillus thermosulfidooxidans* Strain ST

**DOI:** 10.1371/journal.pone.0099417

**Published:** 2014-06-18

**Authors:** Xue Guo, Huaqun Yin, Yili Liang, Qi Hu, Xishu Zhou, Yunhua Xiao, Liyuan Ma, Xian Zhang, Guanzhou Qiu, Xueduan Liu

**Affiliations:** 1 School of Minerals Processing and Bioengineering, Central South University, Changsha, China; 2 Key Laboratory of Biometallurgy of Ministry of Education, Central South University, Changsha, China; Missouri University of Science and Technology, United States of America

## Abstract

The genus *Sulfobacillus* is a cohort of mildly thermophilic or thermotolerant acidophiles within the phylum *Firmicutes* and requires extremely acidic environments and hypersalinity for optimal growth. However, our understanding of them is still preliminary partly because few genome sequences are available. Here, the draft genome of *Sulfobacillus thermosulfidooxidans* strain ST was deciphered to obtain a comprehensive insight into the genetic content and to understand the cellular mechanisms necessary for its survival. Furthermore, the expressions of key genes related with iron and sulfur oxidation were verified by semi-quantitative RT-PCR analysis. The draft genome sequence of *Sulfobacillus thermosulfidooxidans* strain ST, which encodes 3225 predicted coding genes on a total length of 3,333,554 bp and a 48.35% G+C, revealed the high degree of heterogeneity with other *Sulfobacillus* species. The presence of numerous transposases, genomic islands and complete CRISPR/Cas defence systems testifies to its dynamic evolution consistent with the genome heterogeneity. As expected, *S. thermosulfidooxidans* encodes a suit of conserved enzymes required for the oxidation of inorganic sulfur compounds (ISCs). The model of sulfur oxidation in *S. thermosulfidooxidans* was proposed, which showed some different characteristics from the sulfur oxidation of Gram-negative *A. ferrooxidans*. Sulfur oxygenase reductase and heterodisulfide reductase were suggested to play important roles in the sulfur oxidation. Although the iron oxidation ability was observed, some key proteins cannot be identified in *S. thermosulfidooxidans*. Unexpectedly, a predicted sulfocyanin is proposed to transfer electrons in the iron oxidation. Furthermore, its carbon metabolism is rather flexible, can perform the transformation of pentose through the oxidative and non-oxidative pentose phosphate pathways and has the ability to take up small organic compounds. It encodes a multitude of heavy metal resistance systems to adapt the heavy metal-containing environments.

## Introduction

In extremely acidic environments such as acid mine drainage (AMD), low pH, high toxic element concentrations and low levels of organic materials make growth conditions harsh. While these conditions are toxic for most of prokaryotic and eukaryotic organisms, some bacteria and archaea are not only resistant to but also able to metabolize the toxic compounds present [1]. Members of the *Sulfobacillus* genus are typical examples and frequently occur in AMDs, acid hot springs, and hydrothermal vents, as *Sulfobacillus benefaciens* [2] and *Sulfobacillus acidophilus* [3]. Taxonomically, the genus *Sulfobacillus*, along with the genus *Thermaerobacter*, have tentatively been assigned to a family, “*Clostridiales* family XVII *incertae sedis*”, which may form a deep branch within the phylum *Firmicutes* or may form a new phylum [4]. Until now, five species have been isolated and assigned to the genus *Sulfobacillus* [5], all of which are mildly thermophilic or thermotolerant acidophiles, which grow optimally in mixotrophic media containing inorganic sulfur compounds (ISCs), mineral sulfides and organic matters [6]. Some of these species that have been tested also have the ability of iron oxidation [3], [7].

Over the past few years, an increasing number of acidophile genomes have been sequenced. There are now at least 56 draft or completely sequenced genomes of acidophilies including 30 bacteria and 26 archaea, providing a first glimpse of the genomics of acidophilic life over a range of environmental conditions [8]. However, most of these genomes belong to Gram-negative acidophiles, till today only *S. acidophilus* in the genus *Sulfobacillus* has been investigated genomically, revealing that *Sulfobacillus* exploits a surprisingly different enzymatic repertoire for energy and carbon metabolism compared with the *Acidithiobacillus* counterparts. For example, *S. acidophilus* lacks homologues for rusticyanin typically found in iron-oxidizing acidophilies [9], suggesting different components for electron transport in the iron oxidation. Furthermore, members of the genus *Sulfobacillus* also exhibit remarkably different abilities of iron (II) oxidation and sulfide oxidation [10]. However, a comprehensive understanding of the metabolic versatility and environmental adaptations of *Sulfobacillus* will require comparative genomic analysis with more members of the genus *Sulfobacillus*.

In this study, *S. thermosulfidooxidans* strain ST isolated from an acid hot spring in Tengchong, Yunnan (Southwestern China) presents interesting physiological and metabolic capacities (Fig. S1 in File S1 and Table 1). A deep coverage draft genome of *S. thermosulfidooxidans* strain ST was sequenced and analyzed. It was next compared to the genomes of other thermophiles to explore the physiology of *S. thermosulfidooxidans* at the whole genome level. Furthermore, semi-quantitative RT-PCR analysis was performed to verify the expressions of key genes related with iron and sulfur oxidation. A detailed analysis of energy metabolism and central carbon metabolism in *S. thermosulfidooxidans* was described. This genome exploration revealed a very dynamically evolving genome contributing to an unexpected physiological versatility.

**Table 1:** General features of the *S. thermosulfidoxidans* genome in comparison with other *Clostridiales* family XVII *incertae sedis* genomes.

Organism	*Sulfobacillus thermosulfidooxidans*ST	*Sulfobacillus acidophilus*DSM 10332	*Sulfobacillus* *acidophilus*TPY	*Thermaerobacter* *arianensis* DSM12885
Habitat	Acid hot spring	Acidic sulfidicand sulfuroussites	Hydrothermalvent in thePacific Ocean	Mud from theMariana Trench inthe Pacific Ocean
Temperature range	Moderatethermophilic (48°C optimum)	Moderate thermophilic (50°Coptimum)	Moderatethermophilic(Approximately 50°C)	Thermophilic (50–80°C)
pH range	1.2–2.4	1.6–2.3	1.6–2.3	5.4–9.5
Motility	Non-motile	Non-motile	motile	Non-motile
Nutrition type	Mixotrophic	Mixotrophic	Mixotrophic	Chemoheterotroph
Genome size in Mb	3.33	3.56	3.55	2.84
G+C content	48.35%	56.8%	56.8%	72.5%
Protein-coding genes	3225	3585	3837	2435
16S-23S rRNA genes	1	5	5	2
Number of tRNAs	48	69	52	60

## Materials and Methods

### Growth of *S. thermosulfidooxidans* Strain ST and DNA Extraction

*Sulfobacillus thermosulfidooxidans* strain ST was grown at 45°C aerobically in 9 K basal salt medium [11] with 4.5% (w/v) ferrous sulfate. Microbial cells were harvested by centrifugation (12,000 rmp) for 10 min at 4°C. Genomic DNA was extracted from the pelleted cells using TIANamp Bacteria DNA kit (TIANGEN, China) according to the manufacturer’s instruction and finally suspended in MilliQ water. The genomic DNA was stored in −80°C until used for genome sequencing.

### RNA Extraction and Semi-quantitative RT-PCR Analysis

45 g/L ferrous sulfate and 10 g/L elemental sulfur (S^0^) were separately used as substrate in the cultivation. Microbial cells were harvested in mid exponential growth phase. Total RNA was extracted using TRIzol reagent (Invitrogen, Carlsbad, USA), treated with RNase-free DNase I (Qiagen, Valencia, USA) and purified with a RNeasy kit (Qiagen, Valencia, USA). Then, single-stranded cDNA was synthesized with ReverTra Ace qPCR RT Kit (Toyobo, Japan), according to the manufacturer’s protocol. The cDNA was stored at −80°C until used for semi-quantitative RT-PCR analysis.

Primers targeting selected genes putatively involved in ISC metabolism and iron oxidation were designed for semi-quantitative RT-PCR (Table S1 in File S1). The semi-quantitative RT-PCR was performed in 25 µl reaction mixture containing 12.5 µl universal *Taq* PCR Master Mix (Tiangen Biotech, China), 0.5 µl single-stranded cDNA, and 1 µl each of 10 µM forward and reverse primers, and 10 µl deionized water. The specific amplification protocol was as follows: 95°C for 5 min, then 40 cycles of 95°C for 20 s, 55°C for 15 s, and 72°C for 15 s and a final incubation of 72°C for 10 min. PCR products were visualized on 2% agarose gels and sequenced bidirectionally.

### Genome Sequencing, Assembly, Annotation

Genomic library construction, sequencing, and assembly were performed at the Beijing Genomics Institute (BGI; Shenzhen, China) using Illumina Hiseq 2000 sequencing platform and yielded approximately 350 Mb sequence information. Finally, the raw reads were assembled into 53 supercontigs using SOAPdenovo package [12]. Coding sequences (CDSs) were predicted with the ORF finders Glimmer [13]. All CDSs were manually curated and verified by comparison with the publicly available databases NCBI non-redundant[14], KEGG[15], COG[16] using the annotation software BLAST[17]. The unassigned CDSs were further annotated using the hmmpfam program of the HMMER package 26.0 [18]. The hidden Markov models for the protein domains were obtained from the Pfam database 26.0. And the identifications of tRNA and rRNA were performed using the tRNAscan-SE [19] and RNAmmer programs [20], respectively. CRISPR loci were identified using CRISPRFinder [21]. Transporter gene annotations were performed by additionally taking into account the information of transporter classification database [22]. Detailed information on the genes ordered by functional category was summarized in Tables S2, S3, S4, and S5 in File S1. The whole genome shotgun project has been deposited at DDBJ/EMBL/GenBank under the accession number PRJNA203261.

### Genome Synteny Comparisons

Pairwise alignments for dot plot representations were performed on six-frame amino acid translation of the genome sequences of *Sulfobacillus thermosulfidooxidans*, *Sulfobacillus acidophilus* and *Thermaerobacter marianensis* using the Promer program in the MUMmer 3.23 package [23]. The default parameters in all analyses were applied, so exact matches longer that six amino acids were identified and adjacent exact matches were joined if a gap no longer than 30 amino acids occurred. And the resulting clusters were further processed if their matches were longer than 20 amino acids and then aligned using a BLOSUM62 amino acid substitution matrix. Furthermore, the GGDC-Genome-to-Genome Distance Calculator was used to estimate the overall similarity among the three genomes. The inferred digital DNA-DNA hybridization values were calculated [24].

### Calculation of COGs for Venn Diagrams

The predicted proteome sequences of selected genomes (*S. acidophilus* and *T. marianensis*) except for *S. thermosulfidooxidans* were retrieved from the NCBI database. The best sequence similarities were obtained by BLAST against COG database using maximal E-value = 1e^−5^. Proteins that were not group into COGs were represented as specific proteins for each organism. The calculation of COGs was performed and visualized with the R package [25].

### Phylogenetic Analyses

Predicted amino acid sequences of selected genes were aligned with reference sequences using multiple sequence alignment tool ClustalW 2.0 [26]. If not mentioned otherwise, phylogenetic trees were constructed using Molecular Evolutionary Genetics Analysis 4.0 software (MEGA, version 4.0).

## Results and Discussion

### Sequencing and Automatic Annotation of the Strain ST Genome

**Figure 1:**
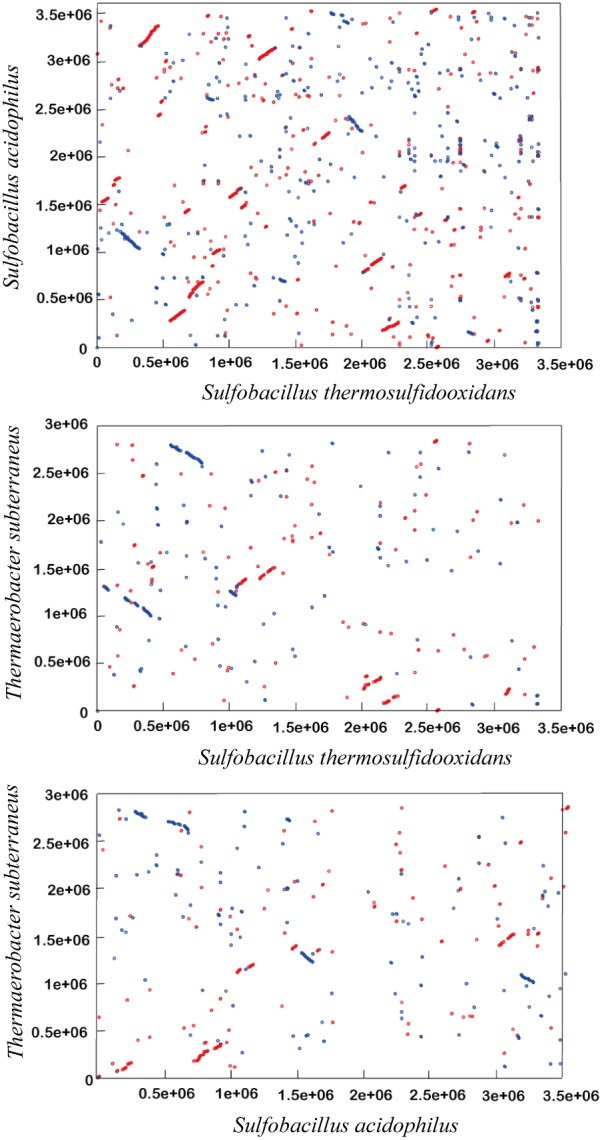
Dot plot representation of the pairwise alignments of the *S. thermosulfidooxidans*, *S. acidophilus* and *T. marianensis* genomes. Alignments were performed on the six-frame amino acid translation of genome sequences using the Promer program in the MUMmer package. In all plots, every dot indicates a match at least six AA between the two genome sequences being compared, with forward matches colored in red and reverse matches colored in blue.

The draft genome sequence of *S. thermosulfidooxidans* strain ST contained 53 supercontigs, ranging from 507 bp to 547,747 bp (the N50 and N90 contig sizes are 376,394 bp and 36,184 bp, respectively), with a total length of 3,333,554 bp. Since most contigs end with repeated sequences, further assembly was not possible with current data. However, given that the present draft has 100× sequence coverage, it is reasonable to assume that the majority of genes in genome of the strain ST are identified from the current draft. It differs from those members of the *Clostridiales family XVII incertae sedis* by its lower overall G+C content (48.35%) (Table 1). Furthermore, within 3225 predicted open reading frames (ORFs), 704 ORFs (22%) are annotated as hypothetical proteins and more than 1190 predicted proteins of *S. thermosulfidooxidans* do not have the best hits within the members of *Clostridiales* family *XVII incertae sedis*. Likewise, few larger syntenic regions were observed between the *S. thermosulfidooxidans* genome and those of other *Clostridiales* family *XVII incertae sedis* (Fig. 1). The inferred digital DNA-DNA hybridization value by the GGDC-Genome-to-Genome Distance Calculator for *S. thermosulfidooxidans* and *S. acidophilus* is <30%. Altogether, these findings suggest that the gene complement of *S. thermosulfidooxidans* is significantly different from those of other *Clotridiales* family *XVII incertae sedis*. Furthermore, we also performed a comparative COG analysis among *S. thermosulfidooxidans*, *T. marianensis* and *S. acidophilus* (Fig. S2 in File S1). In general, *S. thermosulfidooxidans* shared more orthologous genes with *S. acidophilus* than *T. marianensis*. But more than one third of genes in *S. thermosulfidooxidans* genome are unique, which suggests that some physiological features of *S. thermosulfidooxidans* differ a lot from the other two bacteria. Besides, one rRNA operon containing 16S, 23S and 5S rRNA genes was found in the draft genome of *S. thermosulfidooxidans* strain ST (Table S2 in File S1). The 16S rRNA sequence of *S. thermosulfidooxidans* has 93% and 86% nucleotide identity to the 16S rRNAs of *S. acidophilus* and *T. marianensis*. In addition, *S. thermosulfidooxidans* also encodes a similar collection of information processing genes like the other *Sulfobacillus* members (Table S2 in File S1).

### Integrative Elements, CRISPR Defence System

More than 47 transposase genes with transposase signatures are identified in the *S. thermosulfidooxidans* genome and can be assigned to at least nine different types of transposase (Table S3 in File S1) that are associated with insertion sequence families IS3, IS4, IS21, IS66, IS110, IS200, IS605, IS1477 and ISChy4 [27]. Interestingly, the IS element of *S. thermosulfidooxidans* belonging to group IS605 occurs 14 copies in the genome, but they have best BLAST hits with 11 different genera excluding the genus *Sulfobacillus*. Furthermore, most of these IS elements occur in poorly conserved genomic regions, and several elements appear to have inserted into protein-encoding genes indicating that they significantly contribute to genome evolution. Although twenty-seven transposase genes have best hits within the genus *Sulfobacillus*, the organizations surrounding the IS elements are significantly different from those of *S. acidophilus* indicating that these IS elements markedly changed genomic structure of *S. thermosulfidooxidans*. Except for IS elements, some other genes relative with DNA transposition were also identified in the *S. thermosulfidooxidans* genome (Table S3 in File S1). For example, a site-specific recombinase that can catalyze sequential DNA strand exchange reactions [28] was found in the genome. Besides, a cassette chromosome recombinase gene was also identified in the genome (Table S3 in File S1). This cassette chromosome recombinase typically controls resistance gene transmissions [29]. These site-specific recombinases may also greatly contribute genomic variability and physiological versatility.

Interestingly, CRISPR/Cas (clustered regularly interspaced short palindromic repeats/CRISPR-associated genes) viral defence systems [30], [31], [32] were identified in the S. *thermosulfidooxidans* genome (Table S4 in File S1). Eight *cas* genes orienting in the same direction formed a typical CRISPR/Cas system with 44 spacers in the downstream genomic sequence. According to a recent classification of the CRISPR/Cas systems into three major types (I–III), the CRISPR/Cas systems in *S. thermosulfidooxidans* can be assigned into type I CRISPR/Cas systems that is proposed to function in virus defence by directly targeting DNA [33]. Moreover, six CRISPR-associated *cmr* genes orienting in the opposite direction with *cas* genes, all belonging to the repair-associated mysterious protein (RAMP) superfamily [34], are identified in the immediate upstream of *cas* genes. These *cmr* genes may compose a CRISPR viral defence system with 39 spacers in the further upstream, though the functions of these *cmr* genes are largely unknown [33]. Furthermore, the similarities of these *cmr* genes supported the view that they may be obtained by gene horizon transfer. Besides, one more CRISPR locus is identified in the distant genomic region. But only 5 spacers were found in the region and no *cas* or *cmr* genes were found in the region. Furthermore, putative CRISPR loci in the genomes of *S. acidophilus* and *T. marianensis* were also detected by CRISPRfinder [21]. Although we could also identify associated *cas* genes, which are required for viral defence in the two genomes, associated *cmr* genes were not identified in the two genomes. In contrast to *S. thermosulfidooxidans*, the predicted CRISPR sequences of *S. acidophilus* and *T. marianensis* contain fewer spacer-repeat units. There are only 7 repeat/spacer sequences in *S. acidophilus* [3], and 17 repeat/spacer sequences in *T. marianensis* [35]. Unexpectedly, only 2 spacers in *S. thermosulfidooxidans* show similarities to the spacers in *T. marianensis* genome, and none of spacers show similarity to the spacers in *S. acidophilus* genome.

### Energy Metabolism

**Figure 2:**
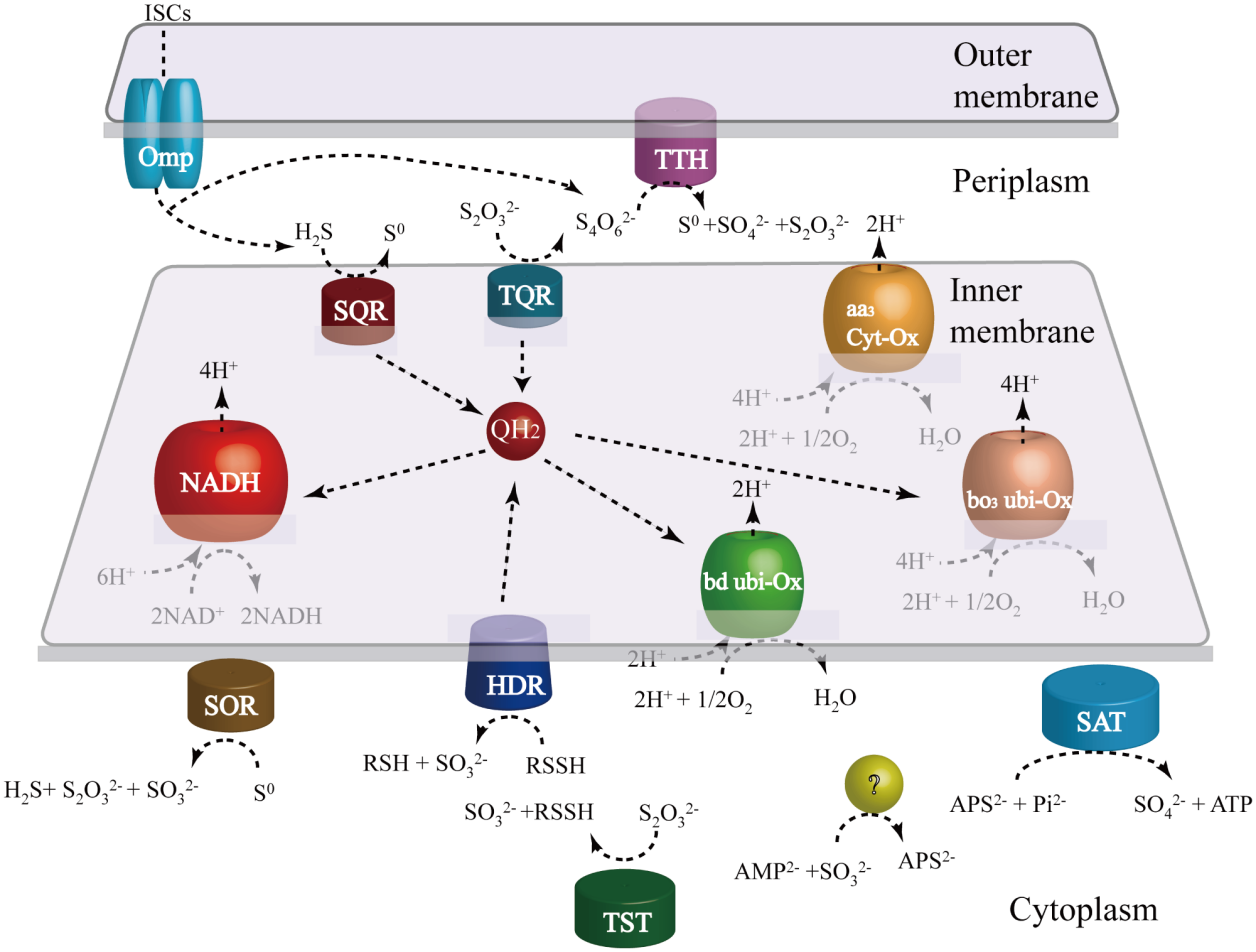
Genome-based models for the oxidation of inorganic sulfur compounds (ISCs). Schematic representation of enzymes and electron transfer proteins involved in the oxidation of ISCs. Electrons from SQR, TQR, HDR are transferred to the electron transfer chain by the quinone, then are used by NADH complex I to generate reducing power or by terminal oxidases *bd* or *bo_3_* to form a proton gradient. Abbreviations: TTH, tetrathionate hydrolase; SQR, sulfide quinone reductase; TQR, thiosulfate quinone oxidoreductase; SOR, sulfur oxygenase reductase; SAT, sulfate adenylyltransferase; HDR, heterodisulfide reductase; Omp, outer membrane protein.

*Sulfobacillus thermosulfidooxidans* can grow mixotrophically by aerobic oxidation of ferrous iron, sulfur, and sulfide in the presence of organic compounds and concomitant fixation of inorganic carbon [36]. The oxidation and electron transfer pathways of ISCs are very complicated and various in different microbes, making their prediction and elucidation difficult [37], [38]. Besides, some steps occur spontaneously, without enzymatic catalysis in the oxidation of ISCs. Previous studies of *S. thermosulfidooxidans* detected several enzymatic activities involved in the oxidation of ISCs, but the specific genes related with these activities were not identified [39], [40]. Based on genome analysis, more than 30 genes encoding enzymes and electron transfer proteins predicted to be involved in the oxidation of inorganic sulfur compounds (ISCs) were detected in the genome (Table S4 in File S1). Genome-based model for ISC metabolism in *S. thermosulfidooxidans* is proposed (Fig. 2). Furthermore, semi-quantitative RT-PCR also indicated that all analyzed genes related with sulfur oxidation are expressed during growth on elemental sulfur.

#### The oxidation of elemental sulfur

**Figure 3:**
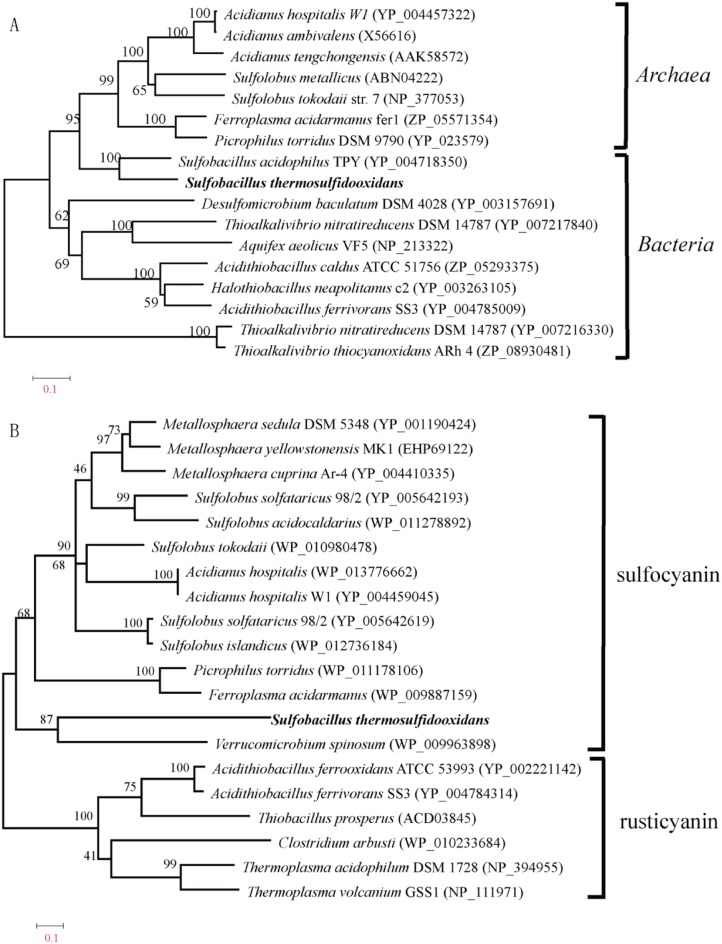
Phylogenetic analysis of key genes related with the oxidation of inorganic sulfur compounds and ferrous iron. (A) Phylogenetic tree of sulfur oxidase reductase based on an alignment of 334 amino acid positions with the neighbor-joining method. The numbers associated with the branches refer to bootstrap values (confidence limits) resulting from 1,000 replicate resamplings. The scale represents the number of amino acid substitutions per site. *S. thermosulfidooxidans* are shown in bold. (B) Phylogenetic tree of sulfocyanin/rusticyanin proteins of archaea and bacteria based on an alignment of 263 amino acid positions with the neighborjoining method. The numbers associated with the branches refer to bootstrap values (confidence limits) resulting from 1,000 replicate resamplings. The scale represents the number of amino acid substitutions per site. *S. thermosulfidooxidans* are shown in bold.

Sulfur oxygenase reductase (SOR) is considered a cytoplasmic enzyme oxidizing elemental sulfur in the cytoplasm in many sulfur-oxidizing bacteria, although it is largely unclear how sulfur is transferred into cytoplasm. It can catalyze substrate sulfur into hydrogen sulfide, sulfite and thiosulfate [41], [42], [43]. In this reaction, elemental sulfur is both the electron donor and one of the two know acceptors, the other being oxygen. The enzyme is different from sulfur dioxygenase [44] and sulfur reductase [45], in that both activities are found together. However, recent study shows that the reaction catalyzed by SOR doesn’t couple with the electron transfer chain or substrate-level phosphorylation in *A. caldus*. The predicted SOR protein in *S. thermosulfidooxidans* shows a characteristic domain of the SOR family (Pfam: PF07682) and all activity-required residues [46]. And the predicted protein shows 72% similarity with the SOR in *S. acidophilus*. Semi-quantitative RT-PCR indicated that the expression of *Sor* gene is very high during growth on elemental sulfur, which suggests that the SOR in *S. thermosulfidooxidans* is also involved in the oxidation of elemental sulfur. Surprisingly, the SORs in *Sulfobacillus* genus are assigned into the archaeal cluster and most closely related to a homologue in archaeal *Ferroplasma acidarmanus* which also survives in extremely acidic environments (Fig. 3.A). These suggest that *Sulfobacillus* genus may exchange the sulfur-oxidizing gene with other extremophiles sharing the similar niche.

One of products from the SOR catalyzed reactions, hydrogen sulfide, is considered to be oxidized by sulfide quinone reductase (SQR). In this reaction, two electrons are transferred to the electron transfer chain by the quinone. In Gram-negative *A. ferrooxidans*, hydrogen sulfide is oxidized by SQR located in the cytoplasmic membrane [37]. One copy of *Sqr* gene was identified in the genome of *S. thermosulfidooxidans*. Meanwhile, an orthologous gene that has the similar gene context to *Sqr* from *S. thermosulfidooxidans* and shares 65% similarity was also identified in *S. acidophilus*. The conservation of *sqr* gene context in the genus *Sulfobacillus* is observed with *A. ferrooxidans*, which strongly suggests that sulfide quinone reductase also has the similar functional properties in Gram-positive *Sulfobacillus* genus.

Another product from the SOR catalyzed reactions, thiosulfate, may be catalyzed by thiosulfate sulfurtransferase (TST) or rhodanese. Five genes encoding thiosulfate sulfurtransferase (TST) or rhodanese are dispersed in *S. thermosulfidooxidans* genome. The presence of the rhodanese motif associated with ubiquitin C-terminal hydrolases and phosphatases makes these enzymes possess the potential of sulfur oxidation[47]. They are proposed to transfer a sulfur atom from thiosulfate to sulfur acceptors like thiol proteins (RSH) with the production of sulfite in *A. caldus* [48]. Subsequently, the product from the TST catalyzed reactions, sulfane sulfate (RSSH), is proposed to be the substrate of heterodisulfide reductase (HDR). heterodisulfide reductase is three subunit complex HdrABC that can catalyze the oxidation of RSSH to regenerate RSH with coupling with the electron transfer chain. Three copies of HdrABC operon have been identified in *S. thermosulfidooxidans* (Table S4 in File S1). One copy of HdrABC operon has a high similarity with that of *S. acidophilus*, while the other two copies of HdrABC operon show more divergences with that of *S. acidophilus*. But the conserved family domains and all activity-required residues [49] were identified in three HdrABC operons. Furthermore, semi-quantitative RT-PCR showed that *HdrABC* genes are highly expressed in growth on elemental sulfur. These results suggest that HDR is also involved in the oxidation of elemental sulfur in the *Sulfobacillus* genus.

#### The sulfite oxidation pathway

The known periplasmic enzymes (sorAB or soxCD) involved in the direct oxidation of sulfite [50], were not identified in both *S. thermosulfidooxidans* and *S. acidophilus*. An oxidoreductase molybdopterin binding protein was identified as a possible sulfite oxidase, but the putative protein only harbors a molybdopterin domain and a twin-arginine translocation pathway signal sequence without dimerization domain and N-terminal heme domain (Table S4 in File S1). Furthermore, no additional subunit containing heme domain that is required for sulfite oxidase was identified in *S. acidophilus* and *S. thermosulfidooxidans*. Therefore, subsequent oxidation of sulfite is most likely to occur in the cytoplasm (Fig. 2). The most possible way for sulfite oxidation is that sulfite is converted to adenosine-5′-phosphosulfate (APS) and then oxidized to sulfate via an indirect pathway controlled by APS reductase and sulfate adenylyltransferase which is similar to the sulfite oxidation pathway in *A. ferrooxidans*. A putative sulfate adenylyltransferase gene was discovered in *S. thermosulfidooxidans*, although no candidates with significant similarity to an orthologous gene of APS reductase were found in the draft genome of *S. thermosulfidooxidans*. An enzyme catalyzing the sulfite to APS is required, if sulfate adenylyltransferase indeed catalyzes APS to sulfate. In *A. ferrooxidans*, the missing function is postulated to be accomplished by the hypothetical gene embedded in the *hdr* locus of sulfur oxidizers [37]. The conserved hypothetical gene was also found in the *hdr* locus of *S. thermosulfidooxidans* genome (Table S4 in File S1).

#### The S4I pathway

The gene encoding tetrathionate hydrolase (TTH) was also identified in *S. thermosulfidooxidans*, which is thought to be involved in the hydrolysis of tetrathionate to generate sulfur, sulfate and thiosulfate. And the activity of this enzyme has been studied in *Acidithiobacillus* genus [52], [53]. The TTH of *S. thermosulfidooxidans* shows 57% similarity with that of *A. caldus* and has a conserved pyrrolo-quinoline quinone domain. Previous experimental data showed that the *A. caldus* TTH is a soluble periplasmic protein with maximum activity at pH 3.0 [54]. Furthermore, *doxDA* genes present in *S. thermosulfidooxidans* genome are predicted to encode a thiosulfate/quinone oxidoreductase (TQR). Orthologous genes of *doxDA* were also detected in *S. acidophilus*,which supports that the enzyme in *Sulfobacillus* genus has the similar functional properties. In our model, the TQR is proposed to catalyze thiosulfate to tetrathionate and transfer two electrons to the quinone. The consecutive reactions catalyzed by TTH and TQR promote the sulfur oxidation in the periplasm of *S. thermosulfidooxidans*.

#### The iron(II) oxidation

In extremely acidic environment, the most detailed account of iron(II) oxidation pathways is available for the Gram-negative bacterium *A. ferrooxidans* [9]. The model for iron oxidation in *A. ferrooxidans* is related to two transcriptional units, the *petI* and *rus* operons [55]. However, the genome of *S. thermosulfidooxidans* doesn’t contain *petI* and *rus* operons, and these genes are not discovered in *S. acidophilus* either. Unexpectedly, a gene encoding the blue copper protein sulfocyanin was found in the *S. thermosulfidooxidans* genome. The predicted protein in *S. thermosulfidooxidans* shows a characteristic domain of sulfocyanin (Pfam: PF06525). Sulfocyanin, sharing sequence characteristics with *A. ferrooxidans* rusticyanins, is proposed to transfer electrons during iron oxidation in acidophilic archaea *Ferroplasma spp*. [56], [57]. Besides, semi-quantitative RT-PCR indicated that the sulfocyanin gene in *S. thermosulfidooxidans* is highly expressed during growth on ferrous sulfate. Thus, it is possible that the predicted sulfocyanin is a component of the electron transport chain in iron oxidation of *S. thermosulfidooxidans*, although more details about iron(II) oxidation pathways are still unknown. Interestingly, phylogenetic analysis revealed that the protein is assigned to the archaeal cluster (Fig. 3.B), which makes the origin of sulfocyanin in *S. thermosulfidooxidans* elusive.

### Electron Transfer Chain

*S. thermosulfidooxidans* encodes a fairly complete respiratory chain consisting of complexes 1–5, which is necessary for energy generation and reverse electron transport (Table S4 in File S1). Three *cydAB* copies encoding subunits of cytochrome *bd* complex and four gene clusters that code for *aa*_3_-type terminal oxidase were detected in the draft genome. The analysis also revealed that *S. thermosulfidooxidans* has 16 genes encoding all subunits of type I NADH dehydrogenase (subunit F of which has three copies). In consistent with other members of the *Sulfobacillus* genus, *S. thernosulfidooxidans* also lacks most components of electron transfer chain in the iron oxidation, only Cyt *aa_3_*, a subunit of the cytochrome complex *bc*_1_ and cytochrome *c* present. However, numerous other genes putatively involved in electron transfer chain were found in *S. thermosulfidooxidans* genome (Table S4 in File S1). The gene redundancy may provide regulatory flexibility to confront environmental changes such as nutrient deficiency and different substrate phosphorylations.

### Central Carbon Metabolism

**Figure 4:**
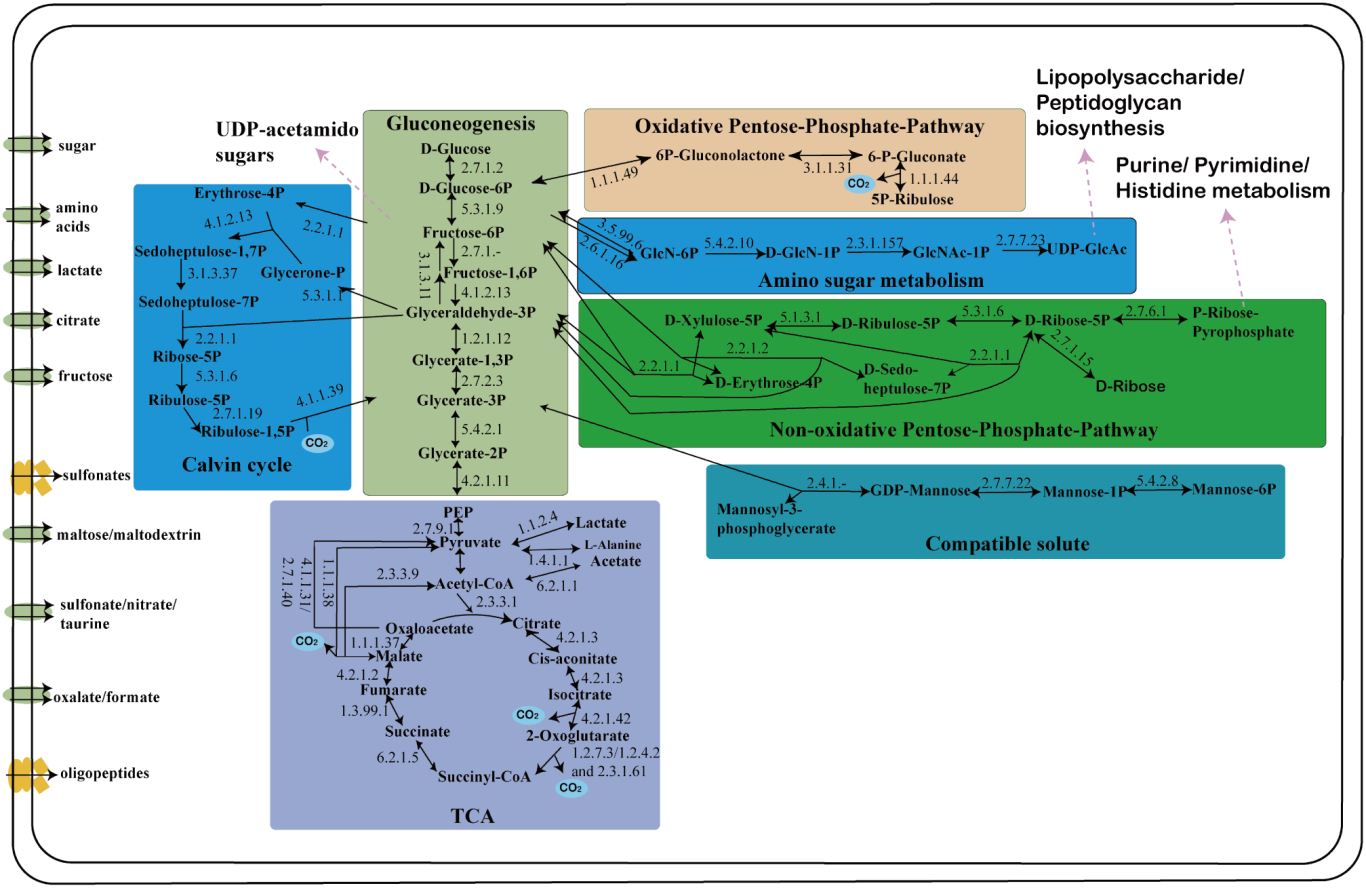
Predicted central carbon metabolism of *S. thermosulfidooxidans* (see also Table S4 in File S1 for further details on respective EC numbers and annotation classification). Enzymatic reactions for which candidate genes can be identified in the genome of *S. thermosulfidooxidans* are highlighted by solid arrows. The reactions associated with other metabolic pathways are shown with pink arrows. The transmembrane transports of small organic compounds that may directly enter central carbon metabolism are also presented.

*S. thermosulfidooxidans* encodes all key genes of the Calvin cycle carbon fixation pathway (Fig. 4; Table S4 in File S1). Comparing with *S. acidophilus* genomes [3], *S. thermosulfidooxidans* only contains a gene cluster encoding form I ribulose bisphosphate carboxylase and lacks homologies of form II ribulose bisphosphate carboxylase. Form I and II ribulose bisphosphate carboxylases are regulated to adapt to environmental conditions with different levels of CO_2_ in *Hydrogenovibrio marinus* [58]. It was reported that *S. thermosulfidooxidans* could grow autotrophically at the CO_2_ content of the supplied air to 5–10% [59]. Thus, the complete Calvin cycle confirms that carbon dioxide can be the major source of carbon for *S. thermosulfidooxidans*.

In many organisms, the 3-phosphoglyceraldehyde generated by CO_2_ fixation via the Calvin cycle enters the glycolysis/gluconeogenesis pathways [47], [60]. The genes identified for the pathways in *S. thermosulfidooxidans* with their reactions and potential interconnections with other biosynthetic pathways are presented (Fig. 4). Fixed carbon can be channeled in either of two directions: for glycogen biosynthesis, or to provide carbon backbones for anabolic reactions. For glycogen biosynthesis, *S. thermosulfidooxidans* contains a gene that are predicted to encode fructose biphosphate aldolase (EC: 4.1.2.13), which catalyzes the formation of fructose-1, 6-bisphosphate. And a fructose biphosphatase gene was identified in *S. thermosulfidooxidans* genome. However, in accordance with all published *Sulfobacillus* genomes, these genomes lack orthologous genes encoding three key enzymes for glycogen biosynthesis: (i) glucose-1P-adenylyltransferase; (ii) glycogen synthase; (iii) 1, 4-alpha-glucan-branching protein. Moreover, *S. thermosulfidooxidans* also lacks glycogen phosphorylase that is involved in decomposing glycogen, thus regenerating glucose-1P from the non-reducing terminus of glycogen [61], [62]. These results suggested that glycogen may not be the main carbon-stored substance in *S. thermosulfidooxidans*.

*S. thermosulfidooxidans* harbours candidate enzymes for all steps of an oxidative TCA (Fig. 4 and Table S4 in File S1). The conversion of 2-oxoglutarate to succinyl-CoA is flexible in *S. thermosulfidooxidans*. It can be catalyzed directly by 2-oxoacid: ferredoxin oxidoreductase (EC: 1.2.7.3) or continuously catalyzed in two steps by 2-oxoglutarate dehydrogenase complex. Moreover, it is notable that *S. thermosulfidooxidans* seems more versatile in the production and the conversion of central metabolite pyruvate (Table S4 in File S1). Except for one copy of malate dehydrogenase (EC: 1.1.1.37), the genome also has two genes with homology to malic enzyme (EC: 1.1.1.38) that catalyzes the reversible conversion of malate to pyruvate and thus is involved in gluconeogenesis and anaplerosis. Furthermore, *S. thermosulfidooxidans* encodes an alanine dehydrogenase (EC: 1.4.1.1) that catalyzes the reversible deamination of alanine to pyruvate (Table S4 in File S1). Alternatively, alanine formation from pyruvate catalyzed by this enzyme might not only be important for protein biosynthesis but could also have a function in ammonia storage and ammonia toxicity alleviation. On the other hand, alanine might thus represent an important source of pyruvate for *S. thermosulfidooxidans*, as it possesses various amino acid and oligo/dipeptide transporters (Table S5 in File S1).

In *S. thermosulfidooxidans*, the process of pentose sugar synthesis is very flexible. *S. thermosulfidooxidans* possesses a functional oxidative pentose phosphate pathway (Fig. 4 and Table S4 in File S1), which can be used for the generation of pentose sugars in many bacteria and archaea [63], [64], [65]. Meanwhile, homologues of key genes for the non-oxidative pentose phosphate pathway could also be identified in *S. thermosulfidooxidans* as well. Specifically, *S. thermosulfidooxidans* encodes a 6-phosphogluconolactonase and two glucose-6-phosphate 1-dehydrogenases that catalyze the interconversion of hexose and pentose (Table S4 in File S1). Furthermore, two genes coding for 6-phosphogluconate dehydrogenase, a key enzyme that bridges the oxidative and non-oxidative part in pentose phosphate pathway, were identified, one of which is located in proximity to two other genes putatively involved in the pentose phosphate pathway, a transketolase and a bifunctional transaldolase/phosoglucose isomerase. The bifunctional transaldolase/phosoglucose isomerase is found as a fused protein harboring one transaldolase domain and one glucose-6-phosphate isomerase domain in many bacteria [66]. It is noteworthy that a gene encodes a mon-functional glucose-6-phosphate isomerase is also identified in the genome (Table S4 in File S1).

### Transport and Resistance

The genome of *S. thermosulfidooxidans* encodes at least 230 putative transporter proteins (Table S5 in File S1), which are the structural elements of approximately 90 transport systems (some of them consisting of several proteins) and represent 50 transporter families[22], [67], [68]. Furthermore, all important components of the general secretion (Sec) and twin-arginine translocation (Tat) pathways have been identified in the genome (Table S4 in File S1). The complement of transport systems in *S. thermosulfidooxidans* is roughly reminiscent of other *Clostridiales* family XVII *incertae sedis* but more than 80 transport proteins have best hits without *Clostridiales* family XVII *incertae sedis*.

Among the *S. thermosulfidooxidans* transport proteins, those belonging to the ATP-binding cassette (ABC) superfamily are the most represented (n = 87), which might be involved in the uptake of organic molecules. Besides, more than 15 transport systems composed of ATP-binding cassette (ABC) superfamily proteins responsible for special organic molecules are also identified. These complement the higher flexibility of *S. thermosulfidooxidans* in the central carbon metabolism. In addition, *S. thermosulfidooxidans* also harbors an oligo/dipeptide transport system as well as several aminopeptidases (leucyl- and methionyl-aminopeptidase), which could be used to release amino acid from the imported peptides. The uptake system of amino acids or peptides may reduce the energy cost of protein synthesis, and could also serve for acquisition of substrates for anaplerotic reactions.

Although the mechanism of transport of extracellular S^0^ to the cytoplasm is not clear, several candidates have been proposed to play important roles for the S^0^ transport in green sulfur bacteria [41], [69]. One possibility is that the thioredoxin SoxW acts together with thiol-disulfide interchange protein DsbD within the periplasm in transferring S^0^ across the inner membrane [70]. One gene is identified that potentially codes for a DsbD protein in the draft genome (Table S5 in File S1). No significant homology of SoxW has been found in *S. thermosulfidooxidans*, however, numerous thioredoxin genes are identified in *S. thermosulfidooxidans* and they possibly can perform the same function as SoxW. Furthermore, *S. thermosulfidooxidans* also possesses at least four sulfonate/nitrate/taurine transport systems. These systems can transport sulfonate compounds into the cytoplasm, and the sulfur in sulfonate compounds may finally be oxidized as energy source.

*S. thermosulfidooxidans* also possesses, like other *Sulfobacillus* members, various resistance systems including more than 30 putative metal ion efflux proteins belonging to 13 different transporter families (Table S5 in File S1). Most of transport systems responsible for metal ion efflux belong to ABC transporter superfamily. Except for these, we also find some oxidoreductases that are closely related with metal resistances (Table S5 in File S1). For example, *S. thermosulfidooxidans* encodes a mercuric reductase, which belongs to a FAD-containing flavoprotein and can reduce Hg^2+^ to Hg^0^ utilizing NADPH [71], [72]. The reduction of Hg^2+^ is an important step of mercuric resistance. As for arsenic resistance, a remarkable feature is the presence of two *arsC* genes coding for arsenate reductase not previously described in *S. thermosulfidooxidans* [73], [74]. The enzyme arsenate reductase is required to confer resistance to As(V) for organisms, since the non-enzymatic reduction of As(V) is too slow to be physiologically significant [75]. And the resulting As(III) can be pumped out of the cells by the ArsA/ArsB ATPase [76]. Surprisingly, phylogenetic analysis showed that one of arsenate reductase is most closely related to a homologue in the thermophilic bacterium *Thermaerobacter subterraneus* but the other is most closely related to a homologue in the acidophilic thermophilic bacterium *Alicyclobacillus acidocaldarius* (Table S5 and Fig. S3 in File S1). These indicate that *S. thermosulfidooxidans* may obtain new arsenic resistance capacities from other extremophiles sharing a similar niche. In addition, *S. thermosulfidooxidans* has three antibiotic transporter systems (Table S5 in File S1), which is absent from other *Sulfobacillus* genomes. It is tempting to speculate that the unique ecological niche makes *S. thermosulfidooxidans* obtain new antibiotic resistances.

## Conclusions

Comparative genome analysis of *S. thermosulfidooxidans* genome revealed that the gene content for sulfur oxidation is similar to other sulfur-oxidizing acidophiles, but also revealed some features not yet found in other acidophiles. A novel sulfur oxygenase reductase is suggested to play a key role in the sulfur oxidation of *S. thermosulfidooxidans*. It can catalyze substrate sulfur into hydrogen sulfide, sulfite and thiosulfate [41], [43]. Although the iron oxidation is still unclear, the predicted sulfocyanin is proposed to be an important component of the electron transport chain in the iron oxidation of *S. thermosulfidooxidans*, as it happens in acidophilic archaea *Ferroplasma spp* [56]. In addition, *S. thermosulfidooxidans* has more flexibility in the central carbon metabolism including two pentose phosphate pathways, flexible conversion of the central metabolite pyruvate and the ability to metabolize various organic compounds. However, glycogen may not be used as a substance of energy source in *S. thermosulfidooxidans*. It also possesses numerous transport systems of organic compounds including multiple sugars, oligopeptide/dipeptide, malic acid, and various amino acids. These transport systems complement the higher flexibility of *S. thermosulfidooxidans* in the central carbon metabolism. Furthermore, it also encodes an impressive collection of resistance proteins that will provide a surviving advantage for living in the acid hot spring containing high concentration of various heavy metals. The physiological versatility of *S. thermosulfidooxidans* might be an essential factor for the competitive success in the extreme acidic environment.

## Supporting Information

File S1 **Figures S1, S2, and S3 and Tables S1, S2, S3, S4 and S5. Figure S1.** A. Phylogeny of *Clostridiales* family XVII *incertae sedis* including *S. thermosulfidooxidans*. Phylogenetic tree based on the 16S rRNA gene was constructed with the neighbor-joining method. The scale bar represents the number of nucleotide substitutions per site. B. Electron micrograph of *S. thermosulfidooxidans* shows its morphology. **Figure S2.** **Venn diagrams showing the numbers of COGs shared between the predicted proteomes of** ***S. thermosulfidooxidans*** **,** ***S. acidophilus*** **, and** ***T. marianensis*** **.** Proteins that were not grouped into COGs are represented as specific proteins for each organism. Numbers in brackets behind species names indicate total number of predicted proteins. **Figure S3.** **Phylogenetic tree of arsenate reductase.** The phylogenetic tree was constructed based on a manually corrected alignment of 159 amino acid positions with the neighbor-joining method. The numbers associated with the branches refer to bootstrap values (confidence limits) resulting from 1,000 replicate resamplings. The scale represents the number of amino acid substitutions per site. The two sequences in *S. thermosulfidooxidans* are shown in bold. **Table S1. Primers used for semi-quantitative RT-PCR. Table S2. Selecting genes encoding informational processing proteins. Table S3. Genes encoding transposase and other enzymes related with gene transposition. Table S4. Genes encoding proteins of diverse metabolic pathways. Table S5. Genes encoding transporter proteins.**
(ZIP)

## References

[1] Johnson DB, Hallberg KB (2003) The microbiology of acidic mine waters. Research in Microbiology 154: 466–473. 14499932 10.1016/S0923-2508(03)00114-1

[2] Johnson DB, Joulian C, d’Hugues P, Hallberg KB (2008) *Sulfobacillus benefaciens* sp. nov., an acidophilic facultative anaerobic *Firmicute* isolated from mineral bioleaching operations. Extremophiles 12: 789–798. 18719854 10.1007/s00792-008-0184-4

[3] Li B, Chen Y, Liu Q, Hu S, Chen X (2011) Complete genome analysis of *Sulfobacillus acidophilus* strain TPY, isolated from a hydrothermal vent in the Pacific Ocean. Journal of bacteriology 193: 5555–5556. 21914875 10.1128/JB.05684-11 PMC3187392

[4] Vos P, Garrity G, Jones D, Krieg NR, Ludwig W, et al. (2009) Bergey’s Manual of Systematic Bacteriology: Volume 3: The Firmicutes: Springer.

[5] Euzéby JP (1997) List of bacterial names with standing in nomenclature: A folder available on the Internet. International Journal of Systematic Bacteriology 47: 590–592. 9103655 10.1099/00207713-47-2-590

[6] Norris PR, Clark DA, Owen JP, Waterhouse S (1996) Characteristics of *Sulfobacillus acidophilus* sp. nov. and other moderately thermophilic mineral-sulphide-oxidizing bacteria. Microbiology 142: 775–783. 8936305 10.1099/00221287-142-4-775

[7] Melamud V, Pivovarova T, Tourova T, Kolganova T, Osipov G, et al (2003) *Sulfobacillus sibiricus* sp. nov., a new moderately thermophilic bacterium. Microbiology 72: 605–612. 14679908

[8] Cárdenas JP, Valdés J, Quatrini R, Duarte F, Holmes DS (2010) Lessons from the genomes of extremely acidophilic bacteria and archaea with special emphasis on bioleaching microorganisms. Applied microbiology and biotechnology 88: 605–620. 20697707 10.1007/s00253-010-2795-9

[9] Bonnefoy V, Holmes DS (2012) Genomic insights into microbial iron oxidation and iron uptake strategies in extremely acidic environments. Environmental Microbiology. 10.1111/j.1462-2920.2011.02626.x 22050575

[10] Shiers D, Ralph D, Watling H (2010) A comparative study of substrate utilisation by *Sulfobacillus* species in mixed ferrous ion and tetrathionate growth medium. Hydrometallurgy 104: 363–369.

[11] Silverman MP, Lundgren DG (1959) Studies on the chemoautotrophic iron bacterium *Ferrobacillus ferrooxidans* I. An improved medium and a harvesting procedure for securing high cell yields. Journal of Bacteriology 77: 642–647. 13654231 10.1128/jb.77.5.642-647.1959 PMC290434

[12] Li R, Yu C, Li Y, Lam TW, Yiu SM, et al (2009) SOAP2: an improved ultrafast tool for short read alignment. Bioinformatics 25: 1966–1967. 19497933 10.1093/bioinformatics/btp336

[13] Delcher AL, Bratke KA, Powers EC, Salzberg SL (2007) Identifying bacterial genes and endosymbiont DNA with Glimmer. Bioinformatics 23: 673–679. 17237039 10.1093/bioinformatics/btm009 PMC2387122

[14] Sayers EW, Barrett T, Benson DA, Bolton E, Bryant SH, et al (2011) Database resources of the national center for biotechnology information. Nucleic acids research 39: D38–D51. 21097890 10.1093/nar/gkq1172 PMC3013733

[15] Ogata H, Goto S, Sato K, Fujibuchi W, Bono H, et al (1999) KEGG: Kyoto encyclopedia of genes and genomes. Nucleic acids research 27: 29–34. 9847135 10.1093/nar/27.1.29 PMC148090

[16] Tatusov RL, Fedorova ND, Jackson JD, Jacobs AR, Kiryutin B, et al (2003) The COG database: an updated version includes eukaryotes. BMC bioinformatics 4: 41. 12969510 10.1186/1471-2105-4-41 PMC222959

[17] Altschul SF, Madden TL, Schäffer AA, Zhang J, Zhang Z, et al (1997) Gapped BLAST and PSI-BLAST: a new generation of protein database search programs. Nucleic acids research 25: 3389–3402. 9254694 10.1093/nar/25.17.3389 PMC146917

[18] Bateman A, Coin L, Durbin R, Finn RD, Hollich V, et al (2004) The Pfam protein families database. Nucleic acids research 32: D138–D141. 14681378 10.1093/nar/gkh121 PMC308855

[19] Lowe TM, Eddy SR (1997) tRNAscan-SE: a program for improved detection of transfer RNA genes in genomic sequence. Nucleic acids research 25: 0955–0964. 10.1093/nar/25.5.955 PMC146525 9023104

[20] Lagesen K, Hallin P, Rødland EA, Stærfeldt H-H, Rognes T, et al (2007) RNAmmer: consistent and rapid annotation of ribosomal RNA genes. Nucleic acids research 35: 3100–3108. 17452365 10.1093/nar/gkm160 PMC1888812

[21] Grissa I, Vergnaud G, Pourcel C (2007) CRISPRFinder: a web tool to identify clustered regularly interspaced short palindromic repeats. Nucleic acids research 35: W52–W57. 17537822 10.1093/nar/gkm360 PMC1933234

[22] Saier MH, Tran CV, Barabote RD (2006) TCDB: the Transporter Classification Database for membrane transport protein analyses and information. Nucleic acids research 34: D181–D186. 16381841 10.1093/nar/gkj001 PMC1334385

[23] Delcher AL, Salzberg SL, Phillippy AM (2003) Using MUMmer to identify similar regions in large sequence sets. Current Protocols in Bioinformatics: 10.13. 11–10 (13): 18. 10.1002/0471250953.bi1003s00 18428693

[24] Auch AF, Klenk H-P, Göker M (2010) Standard operating procedure for calculating genome-to-genome distances based on high-scoring segment pairs. Standards in genomic sciences 2: 142. 21304686 10.4056/sigs.541628 PMC3035261

[25] Chen H, Boutros P (2011) VennDiagram: a package for the generation of highly-customizable Venn and Euler diagrams in R. BMC bioinformatics. 12: 35. 10.1186/1471-2105-12-35 PMC3041657 21269502

[26] Thompson JD, Gibson T, Higgins DG (2002) Multiple sequence alignment using ClustalW and ClustalX. Current Protocols in Bioinformatics: 2.3. 1–2 (3): 22. 10.1002/0471250953.bi0203s00 18792934

[27] Mahillon J, Chandler M (1998) Insertion sequences. Microbiology and Molecular Biology Reviews 62: 725–774. 9729608 10.1128/mmbr.62.3.725-774.1998 PMC98933

[28] Hallet B, Arciszewska LK, Sherratt DJ (1999) Reciprocal control of catalysis by the tyrosine recombinases XerC and XerD: an enzymatic switch in site-specific recombination. Molecular cell 4: 949–959. 10635320 10.1016/s1097-2765(00)80224-5

[29] Katayama Y, Ito T, Hiramatsu K (2000) A new class of genetic element, staphylococcus cassette chromosome mec, encodes methicillin resistance in *Staphylococcus aureus* . Antimicrobial Agents and Chemotherapy 44: 1549–1555. 10817707 10.1128/aac.44.6.1549-1555.2000 PMC89911

[30] Horvath P, Barrangou R (2010) CRISPR/Cas, the immune system of bacteria and archaea. Science 327: 167–170. 20056882 10.1126/science.1179555

[31] Deveau H, Garneau JE, Moineau S (2010) CRISPR/Cas system and its role in phage-bacteria interactions. Annual review of microbiology 64: 475–493. 10.1146/annurev.micro.112408.134123 20528693

[32] Sorek R, Kunin V, Hugenholtz P (2008) CRISPR–a widespread system that provides acquired resistance against phages in bacteria and archaea. Nature Reviews Microbiology 6: 181–186. 18157154 10.1038/nrmicro1793

[33] Makarova KS, Haft DH, Barrangou R, Brouns SJ, Charpentier E, et al (2011) Evolution and classification of the CRISPR–Cas systems. Nature Reviews Microbiology 9: 467–477. 21552286 10.1038/nrmicro2577 PMC3380444

[34] Makarova KS, Grishin NV, Shabalina SA, Wolf YI, Koonin EV (2006) A putative RNA-interference-based immune system in prokaryotes: computational analysis of the predicted enzymatic machinery, functional analogies with eukaryotic RNAi, and hypothetical mechanisms of action. Biology direct 1: 7. 16545108 10.1186/1745-6150-1-7 PMC1462988

[35] Han C, Gu W, Zhang X, Lapidus A, Nolan M, et al (2010) Complete genome sequence of *Thermaerobacter marianensis* type strain (7p75aT). Standards in genomic sciences 3: 337. 21304738 10.4056/sigs.1373474 PMC3035304

[36] Xia J-l, Yang Y, He H, Liang C-l, Zhao X-j, et al (2010) Investigation of the sulfur speciation during chalcopyrite leaching by moderate thermophile *Sulfobacillus thermosulfidooxidans* . International Journal of Mineral Processing 94: 52–57.

[37] Quatrini R, Appia-Ayme C, Denis Y, Jedlicki E, Holmes D, et al (2009) Extending the models for iron and sulfur oxidation in the extreme acidophile *Acidithiobacillus ferrooxidans* . BMC genomics 10: 394. 19703284 10.1186/1471-2164-10-394 PMC2754497

[38] Mangold S, Valdés J, Holmes DS, Dopson M (2011) Sulfur metabolism in the extreme acidophile *Acidithiobacillus caldus*. Frontiers in microbiology 2. 10.3389/fmicb.2011.00017 PMC3109338 21687411

[39] Egorova M, Tsaplina I, Zakharchuk L, Bogdanova T, Krasil’nikova E (2004) Effect of cultivation conditions on the growth and activities of sulfur metabolism enzymes and carboxylases of *Sulfobacillus thermosulfidooxidans* subsp. asporogenes strain 41. Applied Biochemistry and Microbiology 40: 381–387. 15455718

[40] Krasil’nikova E, Bogdanova T, Zakharchuk L, Tsaplina I (2004) Sulfur-metabolizing enzymes in thermoacidophilic bacteria *Sulfobacillus sibiricus* . Applied Biochemistry and Microbiology 40: 53–56.

[41] Friedrich CG, Bardischewsky F, Rother D, Quentmeier A, Fischer J (2005) Prokaryotic sulfur oxidation. Current opinion in microbiology 8: 253–259. 15939347 10.1016/j.mib.2005.04.005

[42] Friedrich CG, Rother D, Bardischewsky F, Quentmeier A, Fischer J (2001) Oxidation of reduced inorganic sulfur compounds by bacteria: emergence of a common mechanism? Applied and Environmental Microbiology 67: 2873–2882. 11425697 10.1128/AEM.67.7.2873-2882.2001 PMC92956

[43] Kletzin A (1992) Molecular characterization of the sor gene, which encodes the sulfur oxygenase/reductase of the thermoacidophilic Archaeum *Desulfurolobus ambivalens* . Journal of Bacteriology 174: 5854–5859. 1522063 10.1128/jb.174.18.5854-5859.1992 PMC207119

[44] Rohwerder T, Sand W (2003) The sulfane sulfur of persulfides is the actual substrate of the sulfur-oxidizing enzymes from *Acidithiobacillus* and *Acidiphilium* spp. Microbiology 149: 1699–1710. 12855721 10.1099/mic.0.26212-0

[45] Laska S, Lottspeich F, Kletzin A (2003) Membrane-bound hydrogenase and sulfur reductase of the hyperthermophilic and acidophilic archaeon *Acidianus ambivalens* . Microbiology 149: 2357–2371. 12949162 10.1099/mic.0.26455-0

[46] Urich T, Gomes CM, Kletzin A, Frazão C (2006) X-ray structure of a self-compartmentalizing sulfur cycle metalloenzyme. Science 311: 996–1000. 16484493 10.1126/science.1120306

[47] Valdés J, Pedroso I, Quatrini R, Dodson R, Tettelin H, et al (2008) *Acidithiobacillus ferrooxidans* metabolism: from genome sequence to industrial applications. BMC genomics 9: 597. 19077236 10.1186/1471-2164-9-597 PMC2621215

[48] Chen L, Ren Y, Lin J, Liu X, Pang X, et al (2012) *Acidithiobacillus caldus* sulfur oxidation model based on transcriptome analysis between the wild type and sulfur oxygenase reductase defective mutant. PloS one 7: e39470. 22984393 10.1371/journal.pone.0039470 PMC3440390

[49] Hamann N, Mander GJ, Shokes JE, Scott RA, Bennati M, et al (2007) A cysteine-rich CCG domain contains a novel [4Fe-4S] cluster binding motif as deduced from studies with subunit B of heterodisulfide reductase from *Methanothermobacter marburgensis* . Biochemistry 46: 12875–12885. 17929940 10.1021/bi700679u PMC3543786

[50] Kappler U, Dahl C (2001) Enzymology and molecular biology of prokaryotic sulfite oxidation1. FEMS microbiology letters 203: 1–9. 11557133 10.1111/j.1574-6968.2001.tb10813.x

[51] Ghosh W, Dam B (2009) Biochemistry and molecular biology of lithotrophic sulfur oxidation by taxonomically and ecologically diverse bacteria and archaea. FEMS microbiology reviews 33: 999–1043. 19645821 10.1111/j.1574-6976.2009.00187.x

[52] Hallberg KB, Dopson M, Lindström EB (1996) Arsenic toxicity is not due to a direct effect on the oxidation of reduced inorganic sulfur compounds by *Thiobacillus caldus* . FEMS microbiology letters 145: 409–414.

[53] Brasseur G, Levican G, Bonnefoy V, Holmes D, Jedlicki E, et al (2004) Apparent redundancy of electron transfer pathways via bc(1) complexes and terminal oxidases in the extremophilic chemolithoautotrophic *Acidithiobacillus ferrooxidans.* . Biochimica Et Biophysica Acta-Bioenergetics 1656: 114–126. 10.1016/j.bbabio.2004.02.008 15178473

[54] Bugaytsova Z, Lindstrom EB (2004) Localization, purification and properties of a tetrathionate hydrolase from *Acidithiobacillus caldus* . European Journal of Biochemistry 271: 272–280. 14717695 10.1046/j.1432-1033.2003.03926.x

[55] Quatrini R, Appia-Ayme C, Denis Y, Ratouchniak J, Veloso F, et al (2006) Insights into the iron and sulfur energetic metabolism of *Acidithiobacillus ferrooxidans* by microarray transcriptome profiling. Hydrometallurgy 83: 263–272.

[56] Tyson GW, Chapman J, Hugenholtz P, Allen EE, Ram RJ, et al (2004) Community structure and metabolism through reconstruction of microbial genomes from the environment. Nature 428: 37–43. 14961025 10.1038/nature02340

[57] Dopson M, Baker-Austin C, Bond PL (2005) Analysis of differential protein expression during growth states of *Ferroplasma* strains and insights into electron transport for iron oxidation. Microbiology 151: 4127–4137. 16339958 10.1099/mic.0.28362-0

[58] Yoshizawa Y, Toyoda K, Arai H, Ishii M, Igarashi Y (2004) CO2-responsive expression and gene organization of three ribulose-1, 5-bisphosphate carboxylase/oxygenase enzymes and carboxysomes in *Hydrogenovibrio marinus* strain MH-110. Journal of Bacteriology 186: 5685–5691. 15317772 10.1128/JB.186.17.5685-5691.2004 PMC516815

[59] Tsaplina I, Krasil’nikova E, Zakharchuk L, Egorova M, Bogdanova T, et al (2000) Carbon metabolism in *Sulfobacillus thermosulfidooxidans* subsp. asporogenes, strain 41. Microbiology 69: 271–276. 10920801

[60] Romano A, Conway T (1996) Evolution of carbohydrate metabolic pathways. Research in microbiology 147: 448–455. 9084754 10.1016/0923-2508(96)83998-2

[61] Johnson L (1992) Glycogen phosphorylase: control by phosphorylation and allosteric effectors. The FASEB journal 6: 2274–2282. 1544539 10.1096/fasebj.6.6.1544539

[62] Gokce E, Franck WL, Oh Y, Dean RA, Muddiman DC (2012) In-depth analysis of the *Magnaporthe oryzae* conidial proteome. Journal of proteome research 11: 5827–5835. 23039028 10.1021/pr300604s PMC3690190

[63] Kato N, Yurimoto H, Thauer RK (2006) The physiological role of the ribulose monophosphate pathway in bacteria and archaea. Bioscience, biotechnology, and biochemistry 70: 10–21. 10.1271/bbb.70.10 16428816

[64] Orita I, Sato T, Yurimoto H, Kato N, Atomi H, et al (2006) The ribulose monophosphate pathway substitutes for the missing pentose phosphate pathway in the archaeon *Thermococcus kodakaraensis* . Journal of Bacteriology 188: 4698–4704. 16788179 10.1128/JB.00492-06 PMC1482999

[65] Soderberg T (2005) Biosynthesis of ribose-5-phosphate and erythrose-4-phosphate in archaea: a phylogenetic analysis of archaeal genomes. Archaea 1: 347–352. 15876568 10.1155/2005/314760 PMC2685555

[66] Hanke T, Nöh K, Noack S, Polen T, Bringer S, et al (2013) Combined Fluxomics and Transcriptomics Analysis of Glucose Catabolism via a Partially Cyclic Pentose Phosphate Pathway in *Gluconobacter oxydans* 621H. Applied and Environmental Microbiology 79: 2336–2348. 23377928 10.1128/AEM.03414-12 PMC3623255

[67] Busch W, Saier MH (2002) The transporter classification (TC) system, 2002. Critical reviews in biochemistry and molecular biology 37: 287–337. 12449427 10.1080/10409230290771528

[68] Saier MH, Yen MR, Noto K, Tamang DG, Elkan C (2009) The transporter classification database: recent advances. Nucleic acids research 37: D274–D278. 19022853 10.1093/nar/gkn862 PMC2686586

[69] Frigaard N-U, Dahl C (2008) Sulfur metabolism in phototrophic sulfur bacteria. Advances in microbial physiology 54: 103–200. 10.1016/S0065-2911(08)00002-7 18929068

[70] Sakurai H, Ogawa T, Shiga M, Inoue K (2010) Inorganic sulfur oxidizing system in green sulfur bacteria. Photosynthesis research 104: 163–176. 20143161 10.1007/s11120-010-9531-2

[71] Fox B, Walsh CT (1982) Mercuric reductase. Purification and characterization of a transposon-encoded flavoprotein containing an oxidation-reduction-active disulfide. Journal of Biological Chemistry 257: 2498–2503. 6277900

[72] Barkay T, Kritee K, Boyd E, Geesey G (2010) A thermophilic bacterial origin and subsequent constraints by redox, light and salinity on the evolution of the microbial mercuric reductase. Environmental Microbiology 12: 2904–2917. 20545753 10.1111/j.1462-2920.2010.02260.x

[73] van der Merwe JA, Deane SM, Rawlings DE (2009) Chromosomal arsenic resistance genes from *Sulfobacillus thermosulfidooxidans* and a demonstration that the genetic diversity of arsB among the *sulfobacilli* is similar to that of their 16S rRNA genes. Advanced Materials Research 71: 171–174.

[74] Van Der Merwe J, Deane S, Rawlings D (2010) The chromosomal arsenic resistance genes of *Sulfobacillus thermosulfidooxidans* . Hydrometallurgy 104: 477–482.

[75] Mukhopadhyay R, Rosen BP (2002) Arsenate reductases in prokaryotes and eukaryotes. Environmental health perspectives 110: 745. 12426124 10.1289/ehp.02110s5745 PMC1241237

[76] Páez-Espino D, Tamames J, de Lorenzo V, Cánovas D (2009) Microbial responses to environmental arsenic. Biometals 22: 117–130. 19130261 10.1007/s10534-008-9195-y

