# Supplementary figures and images for: Comparative Genome Analysis Reveals Metabolic Versatility and Environmental Adaptations of Sulfobacillus thermosulfidooxidans Strain ST
Source: PLoS One. 2014 Jun 18;9(6):e99417. doi: 10.1371/journal.pone.0099417 (PMC4062416; doi:10.1371/journal.pone.0099417)

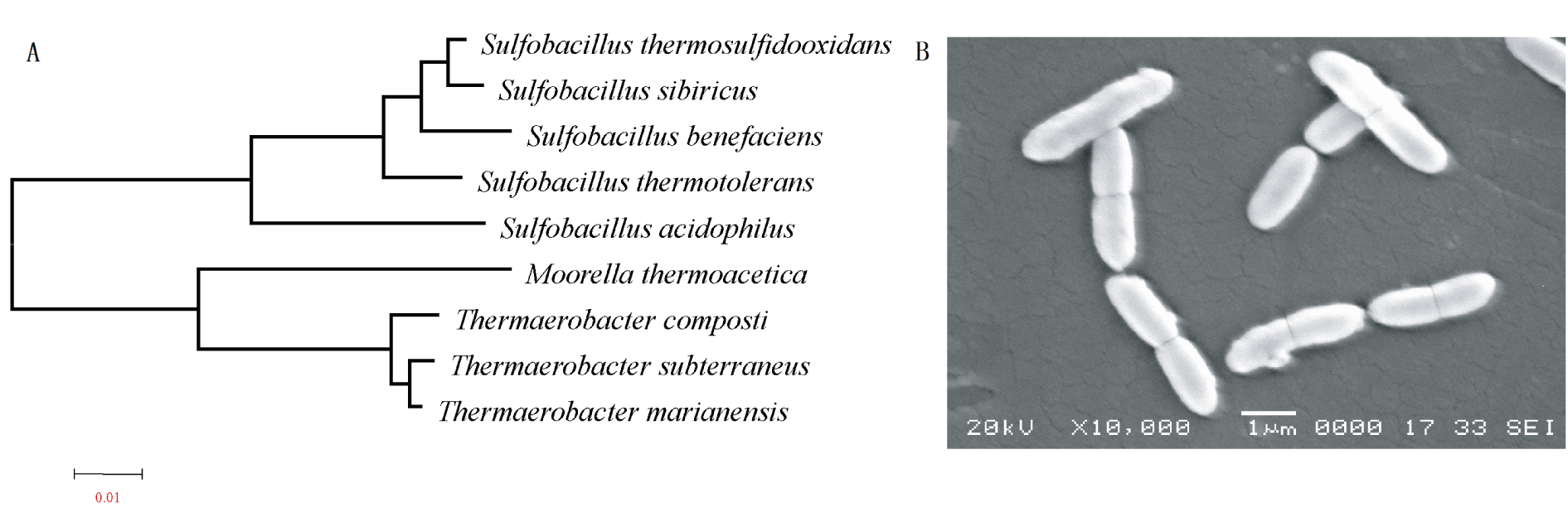

Supplement: File S1 — Figures S1, S2, and S3 and Tables S1, S2, S3, S4 and S5. Figure S1. A. Phylogeny of Clostridiales family XVII incertae sedis including S. thermosulfidooxidans. Phylogenetic tree based on the 16S rRNA gene was constructed with the neighbor-joining method. The scale bar represents the number of nucleotide substitutions per site. B. Electron micrograph of S. thermosulfidooxidans shows its morphology. Figure S2. Venn diagrams showing the numbers of COGs shared between the predicted proteomes of S. thermosulfidooxidans , S. acidophilus , and T. marianensis . Proteins that were not grouped into COGs are represented as specific proteins for each organism. Numbers in brackets behind species names indicate total number of predicted proteins. Figure S3. Phylogenetic tree of arsenate reductase. The phylogenetic tree was constructed based on a manually corrected alignment of 159 amino acid positions with the neighbor-joining method. The numbers associated with the branches refer to bootstrap values (confidence limits) resulting from 1,000 replicate resamplings. The scale represents the number of amino acid substitutions per site. The two sequences in S. thermosulfidooxidans are shown in bold. Table S1. Primers used for semi-quantitative RT-PCR. Table S2. Selecting genes encoding informational processing proteins. Table S3. Genes encoding transposase and other enzymes related with gene transposition. Table S4. Genes encoding proteins of diverse metabolic pathways. Table S5. Genes encoding transporter proteins. (ZIP) [file pone.0099417.s001.zip › Fig.S1.tif]

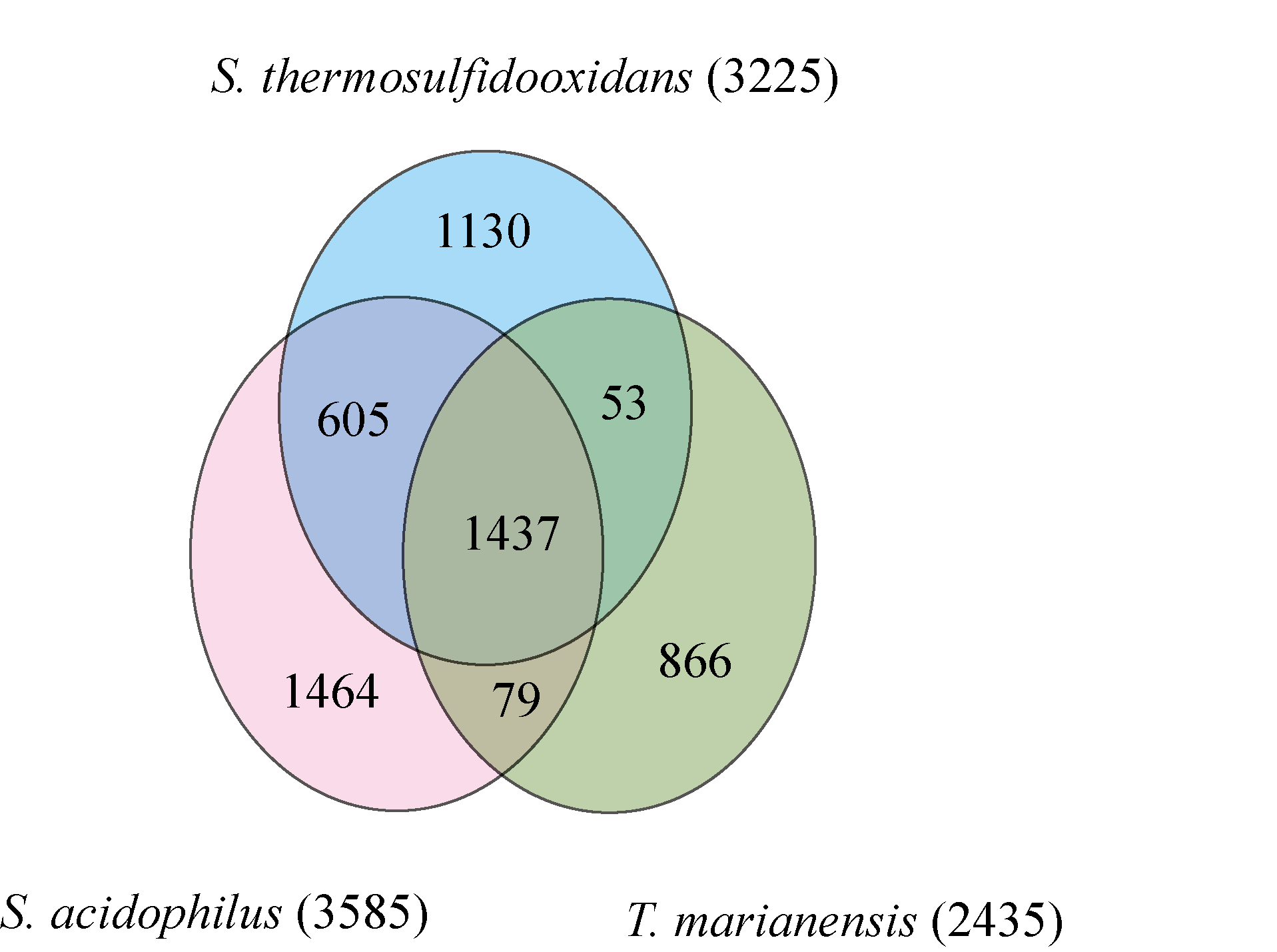

Supplement: File S1 — Figures S1, S2, and S3 and Tables S1, S2, S3, S4 and S5. Figure S1. A. Phylogeny of Clostridiales family XVII incertae sedis including S. thermosulfidooxidans. Phylogenetic tree based on the 16S rRNA gene was constructed with the neighbor-joining method. The scale bar represents the number of nucleotide substitutions per site. B. Electron micrograph of S. thermosulfidooxidans shows its morphology. Figure S2. Venn diagrams showing the numbers of COGs shared between the predicted proteomes of S. thermosulfidooxidans , S. acidophilus , and T. marianensis . Proteins that were not grouped into COGs are represented as specific proteins for each organism. Numbers in brackets behind species names indicate total number of predicted proteins. Figure S3. Phylogenetic tree of arsenate reductase. The phylogenetic tree was constructed based on a manually corrected alignment of 159 amino acid positions with the neighbor-joining method. The numbers associated with the branches refer to bootstrap values (confidence limits) resulting from 1,000 replicate resamplings. The scale represents the number of amino acid substitutions per site. The two sequences in S. thermosulfidooxidans are shown in bold. Table S1. Primers used for semi-quantitative RT-PCR. Table S2. Selecting genes encoding informational processing proteins. Table S3. Genes encoding transposase and other enzymes related with gene transposition. Table S4. Genes encoding proteins of diverse metabolic pathways. Table S5. Genes encoding transporter proteins. (ZIP) [file pone.0099417.s001.zip › Fig.S2.tif]

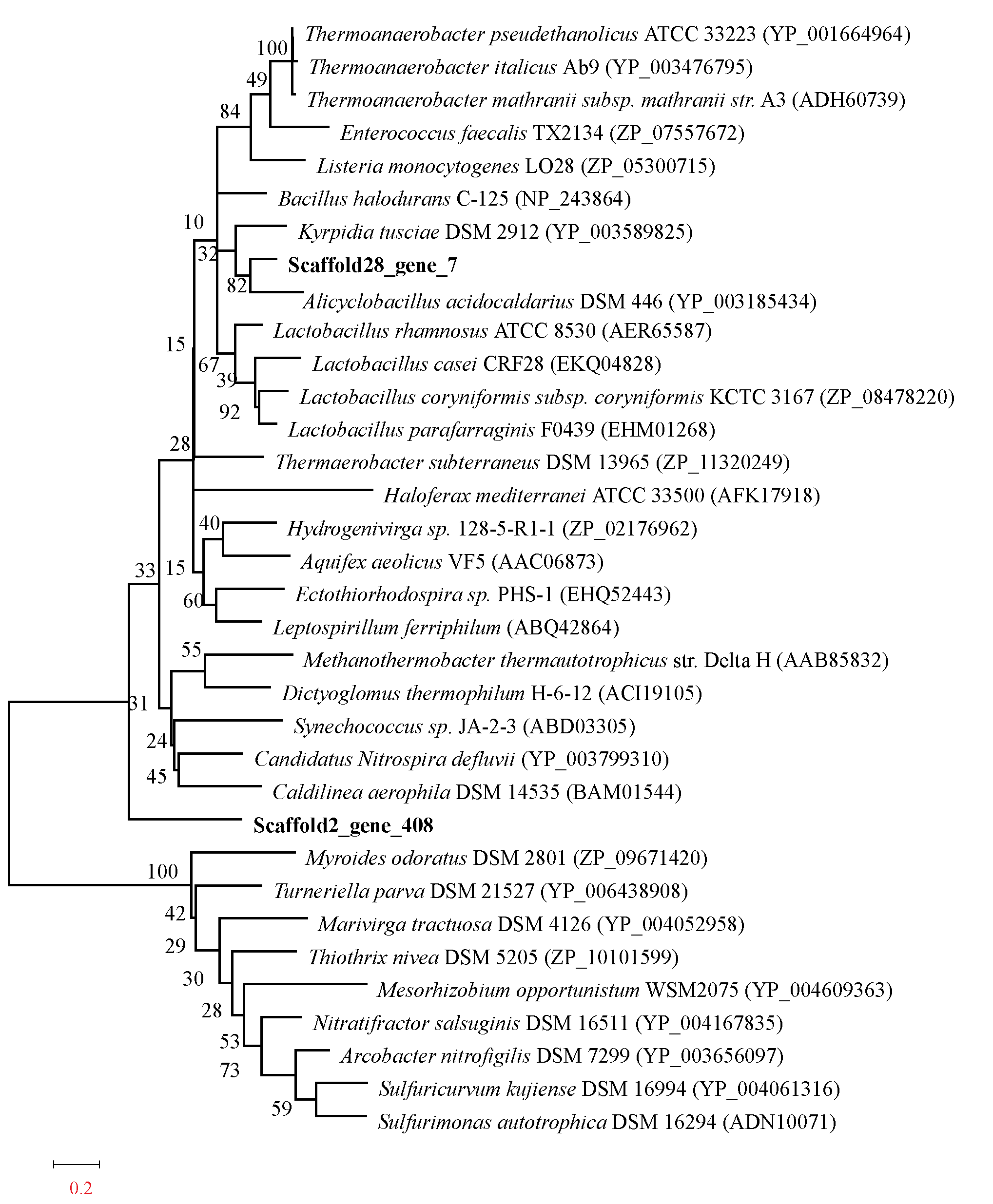

Supplement: File S1 — Figures S1, S2, and S3 and Tables S1, S2, S3, S4 and S5. Figure S1. A. Phylogeny of Clostridiales family XVII incertae sedis including S. thermosulfidooxidans. Phylogenetic tree based on the 16S rRNA gene was constructed with the neighbor-joining method. The scale bar represents the number of nucleotide substitutions per site. B. Electron micrograph of S. thermosulfidooxidans shows its morphology. Figure S2. Venn diagrams showing the numbers of COGs shared between the predicted proteomes of S. thermosulfidooxidans , S. acidophilus , and T. marianensis . Proteins that were not grouped into COGs are represented as specific proteins for each organism. Numbers in brackets behind species names indicate total number of predicted proteins. Figure S3. Phylogenetic tree of arsenate reductase. The phylogenetic tree was constructed based on a manually corrected alignment of 159 amino acid positions with the neighbor-joining method. The numbers associated with the branches refer to bootstrap values (confidence limits) resulting from 1,000 replicate resamplings. The scale represents the number of amino acid substitutions per site. The two sequences in S. thermosulfidooxidans are shown in bold. Table S1. Primers used for semi-quantitative RT-PCR. Table S2. Selecting genes encoding informational processing proteins. Table S3. Genes encoding transposase and other enzymes related with gene transposition. Table S4. Genes encoding proteins of diverse metabolic pathways. Table S5. Genes encoding transporter proteins. (ZIP) [file pone.0099417.s001.zip › Fig.S3.tif]
